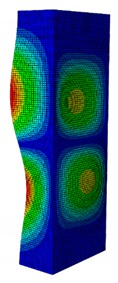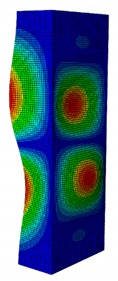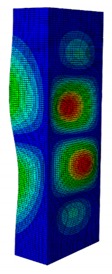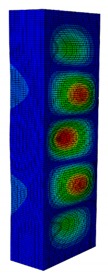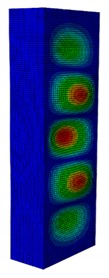# A Numerical Study of the Effect of Component Dimensions on the Critical Buckling Load of a GFRP Composite Strut under Uniaxial Compression

**DOI:** 10.3390/ma13040931

**Published:** 2020-02-19

**Authors:** Quoc Hoan Doan, Duc-Kien Thai, Ngoc Long Tran

**Affiliations:** 1 Department of Civil and Environmental Engineering, Sejong University, 98 Gunja-dong, Gwangjin-gu, Seoul 143-747, Korea; doanquochoan048@gmail.com; 2 Department of Civil Engineering, Vinh University, no.182 Le Duan, Vinh City, Nghe An 43107, Vietnam

**Keywords:** thin-walled structure, GFRP laminated composite, channel section column, buckling behavior, column design curve

## Abstract

In the practical design of thin-walled composite columns, component dimensions should be wisely designed to meet the buckling resistance and economic requirements. This paper provides a novel and useful investigation based on a numerical study of the effects of the section dimensions, thickness ratio, and slenderness ratio on the critical buckling load of a thin-walled composite strut under uniaxial compression. The strut was a channel-section-shaped strut and was made of glass fiber-reinforced polymer (GFRP) composite material by stacking symmetrical quasi-isotropic layups using the autoclave technique. For the purpose of this study, a numerical finite element model was developed for the investigation by using ABAQUS software. The linear and post-buckling behavior analysis was performed to verify the results of the numerical model with the obtained buckling load from the experiment. Then, the effects of the cross-section dimensions, thickness ratio, and slenderness ratio on the critical buckling load of the composite strut, which is determined using an eigenvalue buckling analysis, were investigated. The implementation results revealed an insightful interaction between cross-section dimensions and thickness ratio and the buckling load. Based on this result, a cost-effective design was recommended as a useful result of this study. Moreover, a demarcation point between global and local buckling of the composite strut was also determined. Especially, a new design curve for the channel-section GFRP strut, which is governed by the proposed constitutive equations, was introduced to estimate the critical buckling load based on the input component dimension.

## 1. Introduction

Due to its many advantages, such as low density, high strength, and flexible manufacturing, fiber-reinforced composite materials (e.g., glass fiber-reinforced polymer (GFRP), carbon fiber-reinforced polymer (CFRP), etc.) have been widely used over the past few decades. Nowadays, many industrial fields are applying these materials in areas such as aerospace, aircraft, automotive, and especially civil engineering [1,2,3,4]. In the practical civil engineering field, fiber-reinforced polymer composite materials are often used as profile-type beams or columns. They are applied in various structures, such as bridges, buildings, off-shore structures, etc. [5,6,7]. The profile composite columns represent the thin-walled structures in which their stability is an important factor and is intensely investigated in research and practice. Regarding the stability aspect, the buckling of columns is a complicated problem that often causes the early failure of structures while the yield strength of materials is not reached. Because of its importance, the buckling behavior of columns needs in-depth study. Thus, the buckling of fiber-reinforced composite materials has been widely studied recently [8,9,10,11,12].

To study the structures made of these composite materials for practical use in civil engineering, the experiments often produced various real buckling behavior. Conducting the experiment is a powerful approach for researching new related buckling problems or new types of composite materials [13,14,15]. However, it requires a lot of resources and is often costly [16]. One of the main concerns in the composite industry is to reduce these experimental costs while still obtaining extensive investigation results. Thus, numerical simulations using finite element software have been done to investigate the behavior of these structures [17,18,19]. In this approach, the numerical results are first compared to the experiment results, then numerous extended studies are undertaken based on the developed numerical model.

Due to the complicated structure of composite materials, the modeling task needs to be done carefully to match the experimental results. Moreover, in practical design, extended studies also need to be applicable. A number of numerical models have been developed for the buckling analysis of the GFRP composite materials, especially for the channel-section shape column, such as local buckling and post-buckling behavior [16,20]; progressive failure analysis collapse of the GFRP column [21,22]; buckling, post-buckling, and failure analysis of pre-damaged GFRP column [17,23]; etc. However, in that research, only one certain dimension of the GFRP column is selected for the investigation; some of them considered the change in dimension only through increasing or decreasing the number of laminate layers. As the authors′ acknowledge, the different component dimensions of the GFRP composite column, which is a significant factor in the practical design, has not been studied yet. This factor can contribute to an optimal and applicable design for the practical GFRP column. Moreover, although the channel-section GFRP strut has been studied so extensively, its design equations have never been introduced.

Therefore, this paper introduces a significant numerical study on the effect of the component dimension and slenderness ratio on the critical buckling load and buckling mode of a GFRP composite column. To efficiently perform the investigation, a reliable numerical model was carefully developed by verifying the linear and post-buckling results with the experiment using a finite element software, i.e., ABAQUS^®^ [24]. The effects of the cross-section dimensions, thickness ratio, and slenderness ratio, which are the significant factors in linear buckling analysis, were investigated. A cost-effective design of the GFRP strut is recommended. Especially, a novel design curve and design equations for the channel-section GFRP strut were first proposed to calculate the critical buckling load based on the input dimension of the strut. The investigation found a complex interactive relationship between the component dimensions and the critical buckling load, as well as the mode shape. The results provide beneficial information to designers for making a decision on the stability design of a thin-walled GFRP composite strut.

## 2. Finite Element (FE) Model

### 2.1. Studied Object and Scope

**Figure 1:**
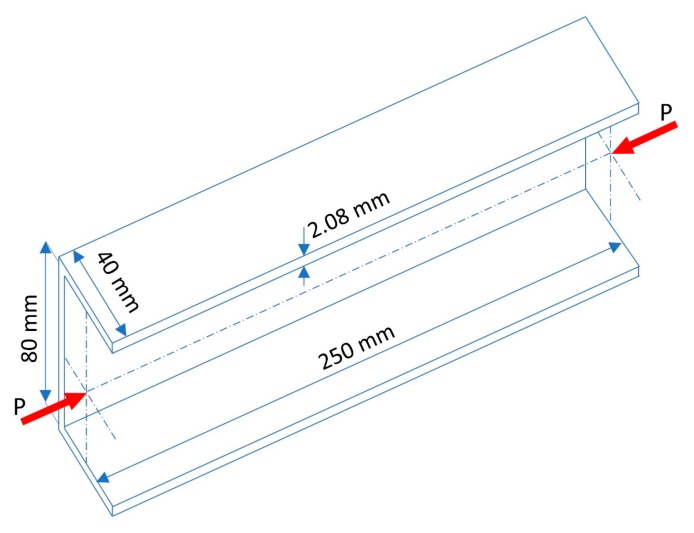
Dimension of the glass fiber-reinforced polymer (GFRP) strut.

In this section, a composite GFRP strut dimension is presented. A uniaxial compression test was conducted by Gliszczynski et al. [25]. The strut was made using an autoclave technique [26,27] and had a channel cross-section shape. It had dimensions of 80 × 40 mm (*B* × *W*) and a length *L* = 250 mm. The thickness of the flanges and the web was equal to 2.08 mm, which was composed of eight 0.26 mm laminated layers. The dimension of the GFRP strut corresponding to the compression test setup is presented in Figure 1.

In this study, the numerical analysis of the GFRP strut was based on a linear buckling analysis in which the critical buckling load was determined by solving an eigenvalue buckling analysis. The results from this analysis were used to investigate the effect of the cross-section dimensions, thickness ratio, and slenderness ratio on the linear buckling load and buckling mode. The accuracy of the FE model was further verified by solving a post-buckling problem with an initialized geometric imperfection. However, this study was limited in terms of investigating the dimension effect of the strut up to the linear critical stage. Thus, the results of the post-buckling analysis can be developed in further study.

### 2.2. Discrete Models of Composite Struts

The finite element method was employed for analyzing the buckling behavior of the thin-walled composite strut using ABAQUS^®^ software. The discretization model of the analyzed column was performed using multi-layered, four-node shell elements (S4R) with six degrees of freedom at each node (three translations and three rotations). The S4R shell stands for a four-node conventional stress/displacement shell element with reduced integration. By using this element, the thin-walled structure geometry is represented using a flat finite element that is degenerated from 3D finite element formulations describing the mid-surface of the structure. To improve the computational efficiency, the plane-stress conditions were applied for the shell element formulation. In the local system (*x*’, *y*’, *z*’) coordinates, the strain field, which is derived from displacement gradient formulations, is expressed as follows [28]:

(1) $$\varepsilon =\nabla \left(\hat{u}\right)={\left[\varepsilon_{x^{\prime }x^{\prime }},\varepsilon_{y^{\prime }y^{\prime }},2\varepsilon_{x^{\prime }y^{\prime }},2\varepsilon_{y^{\prime }z^{\prime }},2\varepsilon_{z^{\prime }x^{\prime }}\right]}^{T},$$

where $\hat{u}$ is the displacement vector within the shell element. The corresponding stress is presented as follows:

(2) $$\sigma ={\left[\sigma_{x^{\prime }x^{\prime }},\sigma_{y^{\prime }y^{\prime }},\tau_{x^{\prime }y^{\prime }},\tau_{y^{\prime }z^{\prime }},\tau_{z^{\prime }x^{\prime }}\right]}^{T}$$

The elastic stress–strain relationship is given by:

(3) $$\sigma =C^{e}\varepsilon$$

where $C^{e}$ is the plane-stress elasticity matrix and is expressed as follows:

(4) $$C^{e}=\frac{E}{1-\nu^{2}}\left[\begin{array}{ccccc} 1 & \nu & 0 & 0 & 0\\ & 1 & 0 & 0 & 0\\ & & \left(1-\nu \right)/2 & 0 & 0\\ & & & k\left(1-\nu \right)/2 & 0\\ sym & & & & k\left(1-\nu \right)/2 \end{array}\right],$$

where *E* and ν denotes Young’s modulus and Poisson′s ratio of the material, respectively; *k* stands for the shear correction factor [29].

**Figure 2:**
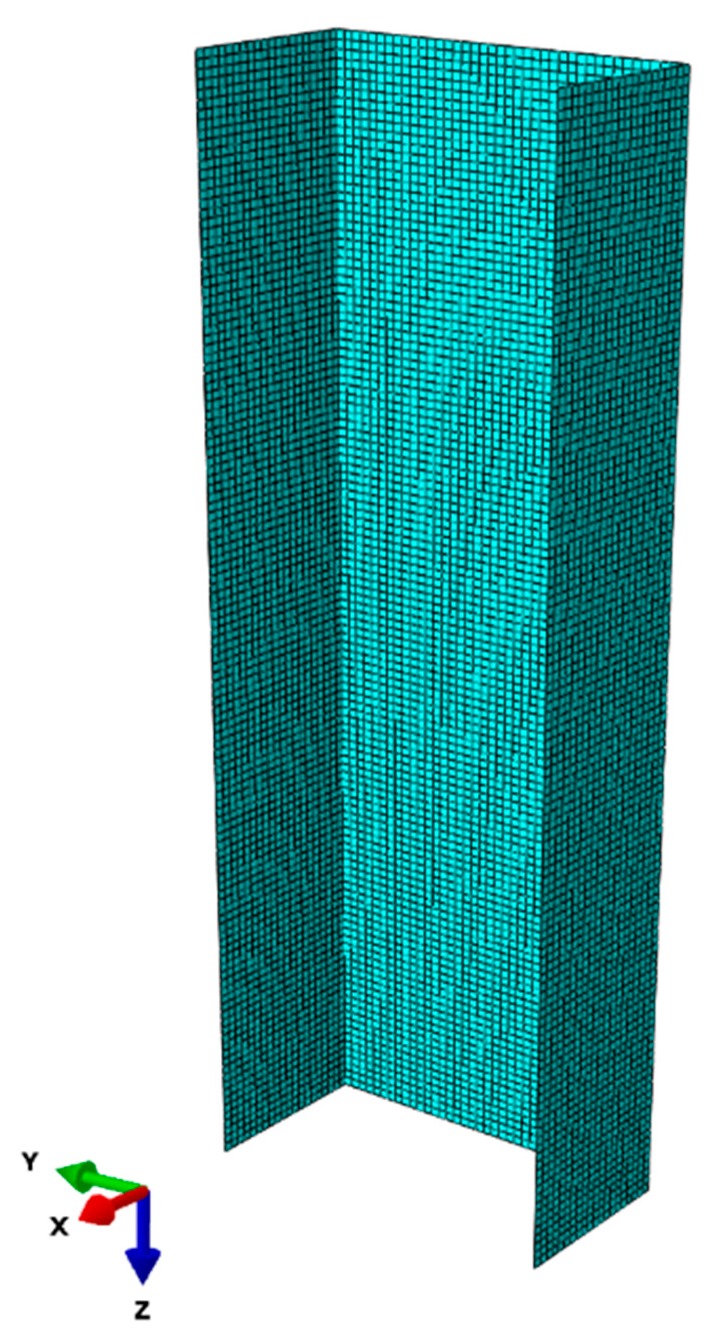
Simulation model of the GFRP strut.

The S4R shell element has shown its suitability for modeling thin-walled composite struts [20,23], thus it was used for modeling the channel section strut in this study. The simulation model of the GFRP composite strut is presented in Figure 2.

The FE numerical model was discretized using a uniform square density in which a single element size was 2 mm. Thus, the flange and web of the strut were composed of 20 and 40 elements, respectively. Furthermore, there were 125 elements along the strut length. This way of discretization ensured a uniform division of the single strut for constituting a constant-density mesh. The mesh size was taken based on the convergence analysis of the thin-walled strut, which was successfully conducted previously [22,30,31]. The convergence analysis provided an effective method to select an element size that achieves a superior accuracy within a reasonable analysis time. In this way, the selected element size may be prevented from making an excessively stiff model when high deflections occur. Moreover, considering the geometry aspect, the 2 mm mesh size allows one to model the boundary conditions generated by the groves more easily.

### 2.3. Model of GFRP Laminate Plate

**Figure 3:**
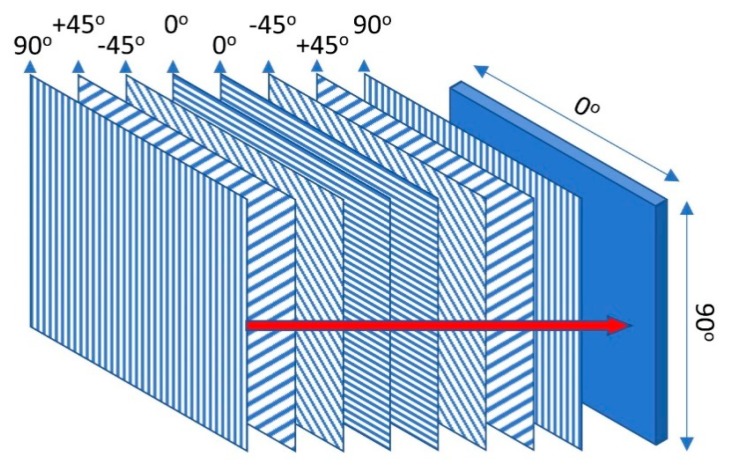
Stacking sequence of a laminate quasi-isotropic plate.

The GFRP laminate plate in this study consisted of eight layers of unidirectional fiber-reinforced laminate. The laminate was defined as a quasi-isotropic material with symmetrical layups of a [0/−45/45/90]s stacking sequence. According to this material, the laminate behaves as an isotropic surface when subjected to in-plane loading. The symmetrical layups can prevent the plate from warping under stress or thermal expansion. The description of the quasi-isotropic laminate layup is presented in Figure 3.

**Figure 4:**
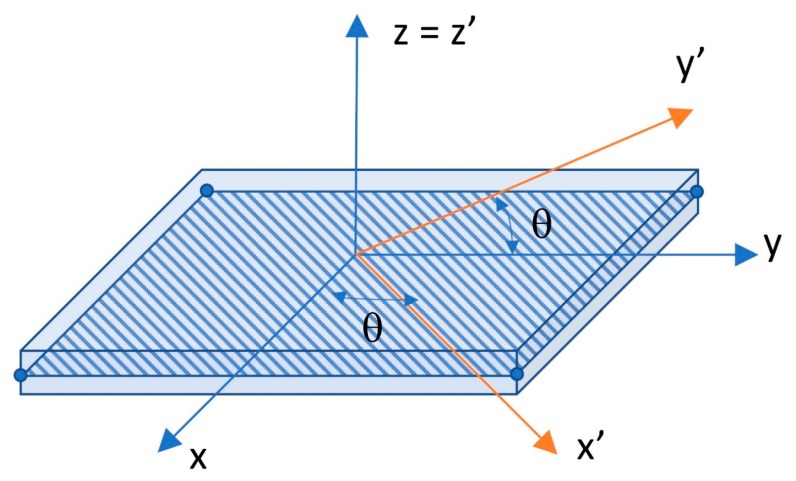
The local coordinate system of a single laminate.

A single laminate can be considered as an orthotropic layer in the ply system (*x*’, *y*’, *z*’), or in other words, the local axis system. The fiber orientation generates an angle θ relative to the global coordinate system of the composite plate and is parallel to its local axis *x*’. The local coordinate system of the laminate is illustrated in Figure 4.

To derive the conditions of the quasi-isotropic laminate, the generalized Hooke′s Law can be applied to define the stress–strain relationship. For an individual lamina isotropic matrix, the extension and shear are ignored and its components are independent of the laminate orientation [32]. The constitutive relation for a plane-stress condition is expressed as:

(5) $$\sigma^{\prime }=Q\varepsilon^{\prime }\Rightarrow \left\{\begin{array}{c} \sigma_{x^{\prime }}\\ \sigma_{y^{\prime }}\\ \tau_{x^{\prime }y^{\prime }} \end{array}\right\}=\left[\begin{array}{ccc} Q_{11} & Q_{12} & 0\\ Q_{21} & Q_{22} & 0\\ 0 & 0 & Q_{66} \end{array}\right]\left\{\begin{array}{c} \varepsilon_{x^{\prime }}\\ \varepsilon_{y^{\prime }}\\ \gamma_{x^{\prime }y^{\prime }} \end{array}\right\},$$

(6) $$\mathrm{with}\;Q_{11}=\frac{E_{1}}{1-v_{12}v_{21}};\;Q_{12}=\frac{v_{12}E_{1}}{1-v_{12}v_{21}};\;Q_{22}=\frac{E_{2}}{1-v_{12}v_{21}};\;Q_{66}=G_{12},$$

where $\sigma^{\prime }$ and $\varepsilon^{\prime }$ are stress and strain vectors in the local coordinate system, respectively; Q denotes the elastic constitutive matrix; $E_{1}$, $E_{2}$, $v_{12}$, $v_{21}$, and $G_{12}$ are the material elastic parameters, where. In the num $v_{21}=v_{12}E_{1}/E_{2}$ erical analysis, the local stress–strain relationship should be transformed into the global coordinate system [16]. The stress in the global system can now be expressed as:

(7) $$\sigma =\bar{Q}\varepsilon =\left(T^{T}QT\right)\varepsilon ,$$

where T is the transformation matrix and is given according to the fiber orientation θ as follows:

(8) $$T=\left[\begin{array}{ccc} \cos^{2}\theta & \sin^{2}\theta & \sin\theta \cos\theta \\ \sin^{2}\theta & \cos^{2}\theta & -\sin\theta \cos\theta \\ -2\sin\theta \cos\theta & 2\sin\theta \cos\theta & \cos^{2}\theta -\sin^{2}\theta \end{array}\right].$$

In this study, the GFRP laminate is composed of plies of uniform thickness. The overall thickness at the web and flange of the strut is identical, which was set to be equal to 2.08 mm. The composite ply thickness corresponding to the fiber orientation is presented in Table 1.

**Figure 5:**
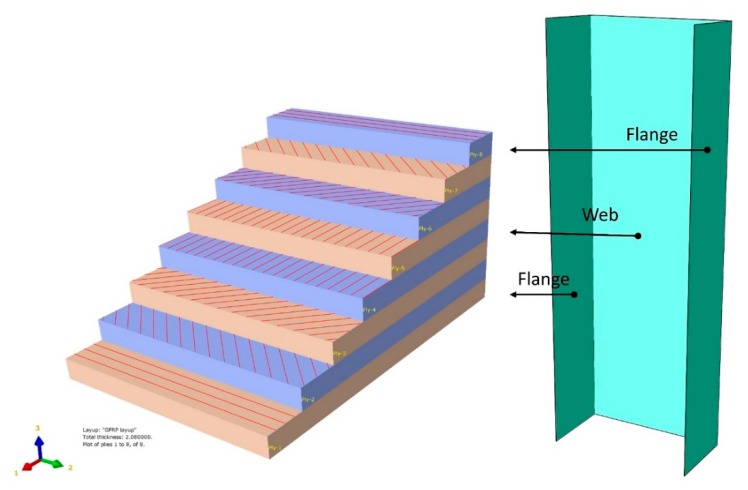
GFRP-laminated plate model in Abaqus.

**Figure 6:**
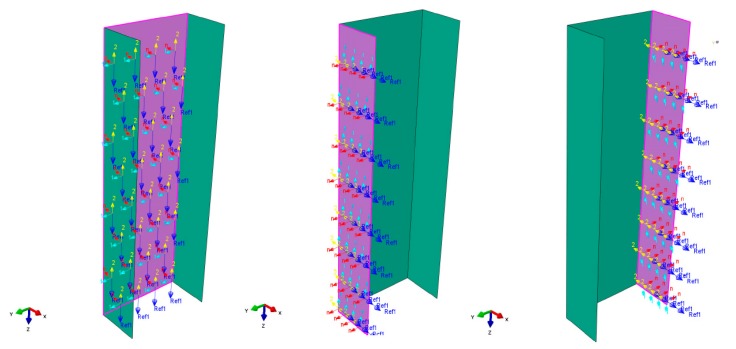
The fiber orientation definition of the web and flanges.

The laminated composite geometry of the GFRP strut was modeled by using the composite layup function in ABAQUS^®^. With the uniform thickness of the strut, the identical properties of composite layers, including the number of plies, ply thickness, and fiber orientations, were assigned to both the web and flange of the strut. The GFRP laminate model and assignment of the fiber orientation are illustrated in Figure 5 and Figure 6, respectively.

### 2.4. Material Properties Model

The material model of the GFRP strut, which was performed in ABAQUS^®^, was based on the assumption used in the previous study of Debski et al. [23]. The mass density of the GFRP material was 2200 (kg/m^3^). The strength properties of the GFRP strut are presented in Table 2.

### 2.5. Model of the Boundary and Loading Conditions

The strut specimen was placed between a top plate and a bottom table. The bottom table, which was mounted to the bottom jaw of a universal testing machine (UTM) produced by Instron (Norwood, MA, USA) and modernized by Zwick-Roel (Ulm, Germany), was a spherical bearing. It included three degrees of freedom that allowed for rotating about three perpendicular axes. The top plate, which was fixed to the upper jaw of the UTM, was installed to transfer the compressive load. It only moved down in the perpendicular direction to the top plate, or in other words, it had only one degree of freedom. To hold the strut specimen vertically, grooves were created on the surface of the plate and table. This generated different boundary conditions on the top and bottom edge of the composite strut. The depth of the grooves was 2 mm. Cylindrical holes in the grooves were milled at the bending corners of the strut to improve fitting. By way of simplification, in this study, only the strut was modeled; the upper and bottom plates were replaced by constraints.

**Figure 7:**
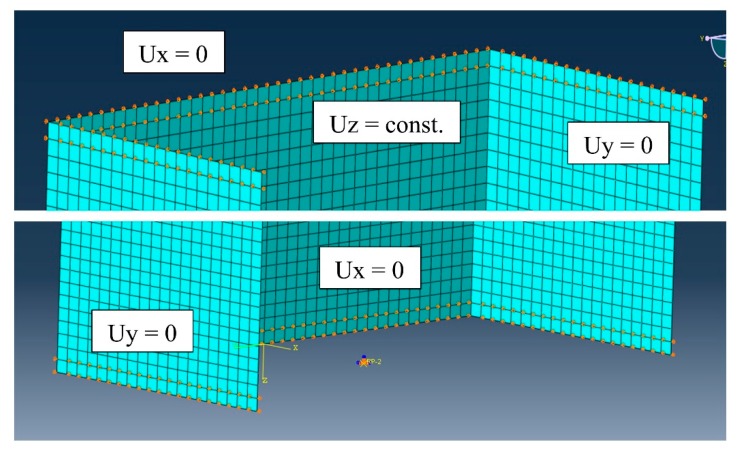
Upper-end and bottom-end boundary conditions of the GFRP strut.

The bottom table was fully fixed by restraining all degrees of freedom at the reference point, while the upper plate could move in the direction of the compressive loading, i.e., along the strut′s longitudinal axis (Z-axis). The shortening of the strut was modeled by the displacement of the upper plate in the Z-axis direction. The ends of the channel section strut were simply supported on the surfaces of rigid plates where contact interactions were defined. The interaction constraints were then assigned, as presented in Figure 7.

The plates were provided with grooves that modeled the shape of a channel section profile, but at the same time, were allowed free rotation on the edges of the strut ends. The simply supported boundary condition was reproduced using a flat-bottomed groove with slightly chamfered edges of 2 mm depth in total. According to the modeling method studied by Banat, Kolakowski, and Mania [33], the grooves were modeled by restraining the ends of the strut with a depth of 2 mm. The bottom table was assumed to be fixed by the jaw of the testing machine. The upper plate was assumed to be constrained in the x and y directions, along with the rotation angle. Its vertical direction z was set to be free. The upper and bottom end boundary conditions of the composite strut is shown in Figure 7.

For the elastic buckling analysis, the linear buckling step was employed using the buckle analysis of linear perturbation procedure in ABAQUS^®^. The GFRP strut was compressed using the UTM with a displacement rate of 1 mm/min. To simulate the vertical compression load generated by the UTM, a uniform load was assigned to a reference point, which represented the upper nodes of the strut by using rigid body-pin (node) constraints. This type of constraint represents the support contact between the strut and the top plate. A nominal compressive load, which was equal to 1.0 N, was assigned to the FE model. The critical buckling load was determined by solving a linear eigenvalue buckling problem. The buckling mode shapes presented in this study were obtained from the first buckling mode, which corresponded to the critical buckling load.

### 2.6. Verification of FE Model

The non-damaged buckling results obtained from the experiment conducted by Gliszczynski et al. [25] were used to verify the proposed FE model in this study. The cross-section dimension and length of the specimen were similar to the modeled FE specimen. To verify the accuracy of the proposed model, the FE buckling behavior results of the quasi-isotropic laminate and angle-ply laminate were compared to the corresponding C1 and C5 specimens of the experiment. The quasi-isotropic laminate (C1) and the angle-ply laminate (C5) had symmetrical layups with [0/−45/+45/90]s and [+45/−45/+45/−45]s stacking sequence, respectively. They were the typical representatives for the GFRP thin-walled material, which were composed of different ply orientations. The comparison of the critical buckling load between the numerical and experiment results is shown in Table 3.

**Figure 8:**
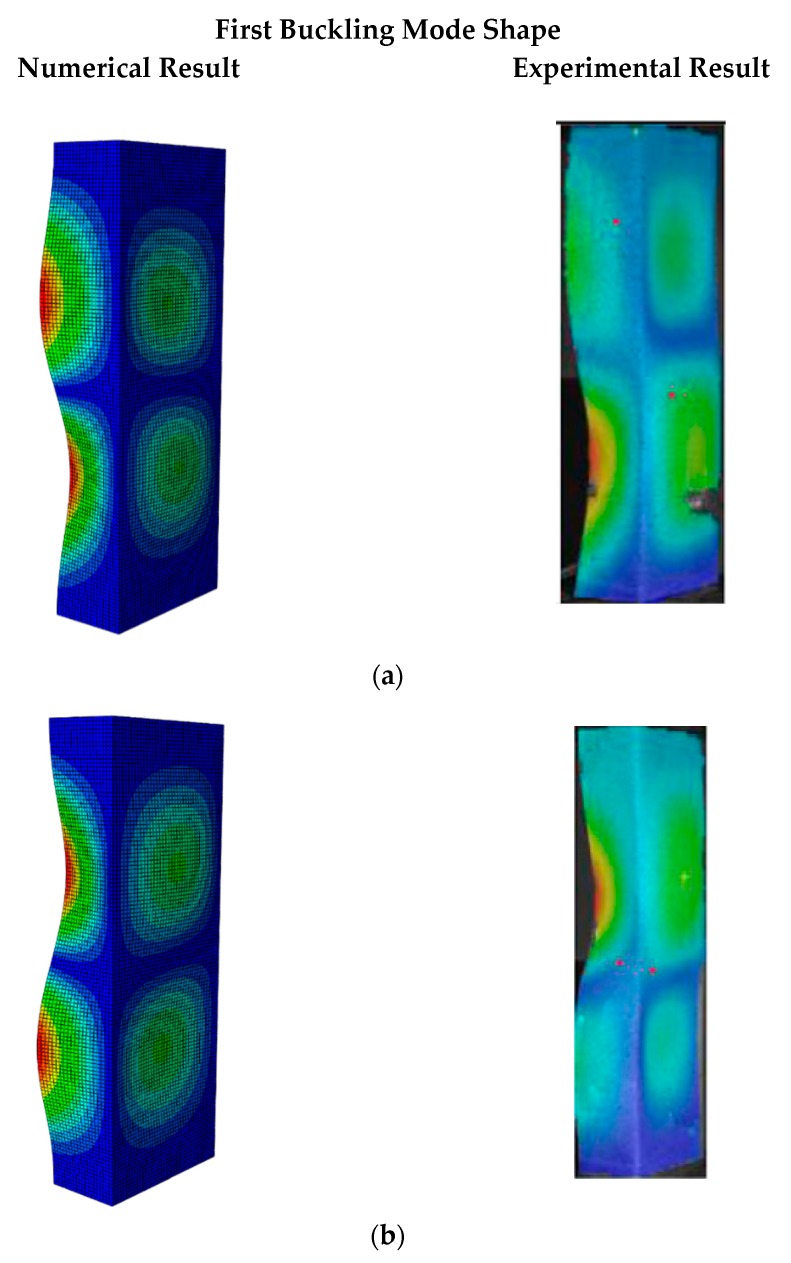
Linear buckling mode shape of the numerical result and experiment result. (**a**) Quasi-isotropic laminate. (**b**) Angle-ply laminate.

As can be seen, the obtained critical buckling load of the quasi-isotropic laminate from the FE model was 11,258 N. Its value was lower than 10% (i.e., allowed error range) of the average buckling load obtained from the experiment. In detail, it was 7.48% greater than the experimental average buckling load of 10,500 N. Similar to the quasi-isotropic laminate, the angle-ply laminate buckling load was 12,652 N, which was 8.14% higher than the experimental result of 11,700 N. The comparison of the first buckling mode shapes between the numerical analysis and the experiment are presented in Figure 8.

As can be seen from Figure 8, the first buckling mode shapes of both the quasi-isotropic laminate and the angle-ply laminate achieved from the proposed FE model were similar to the ones obtained from the experiment. Both the first buckling load and mode shape of the FE model reached a good agreement with the experimental results. The verification results showed that the developed FE model was reliable for further elastic buckling analysis of the composite GFRP strut.

The FE model was further verified by considering the geometric nonlinearity of the GFRP strut. To perform the post-buckling analysis, the buckling load and buckling mode of the previous linear buckling analysis was used as the base state of the strut. According to this, the node displacement obtained from the linear buckling analysis was applied to the initial deformation and the first buckling load was assigned as the initial load for the post-buckling analysis model. The geometric imperfection amplitude of 0.1*h* (i.e., 10% of the strut thickness) was adopted, as introduced by Debski and Jonak [21], since it satisfactorily represents the imperfection rate of the tested channel-section column. Moreover, an additional analysis with different imperfection amplitudes of 0.05*h* and 0.2*h* was also performed to verify the current model.

**Figure 9:**
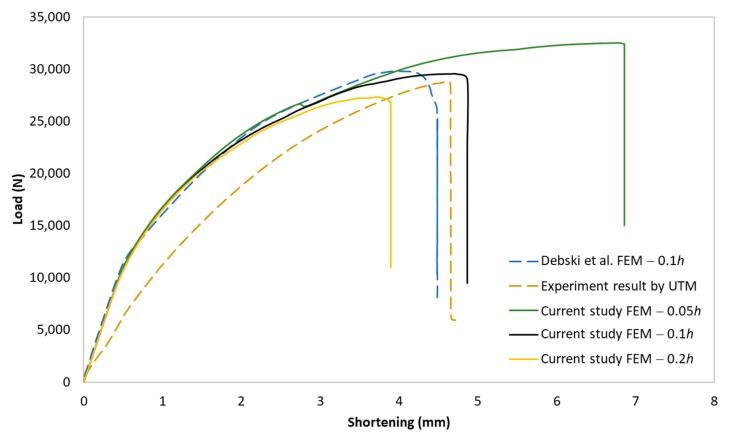
Post-buckling equilibrium paths of the GFRP strut. FEM: finite element model, UTM: universal testing machine.

The post-buckling equilibrium path of the strut in the current study was verified with the result from the experiment done by the UTM and the FE model conducted by Debski et al. [23], as shown in Figure 9. As can be seen, the post-buckling paths of the strut showed a stable bifurcation behavior. The post-critical buckling load of the current study with the imperfection amplitude of 0.1*h* was 29,547 N, while the one obtained from the experiment and the Debski et al. study were around 28,825 N and 29,798 N, respectively. Although the post-buckling paths of the current study and the experiment result included some gaps, it was in high agreement with the path of the FE result in the Debski et al. study. The difference of the post-critical buckling load between the current study and the experiment was about 2.5%, which is less than the tolerance error of 10%. The effect of the geometric imperfection amplitude on the post-critical buckling load was also presented in Figure 9. The results indicate that the post-critical load significantly decreased when the imperfection amplitude increased from 0.05*h* to 0.12*h*. This effect reflected the real behavior of the strut and agreed with the results reported in the literature [16]. These agreements in verification indicate that the current FE model could be utilized for further investigation.

**Figure 10:**
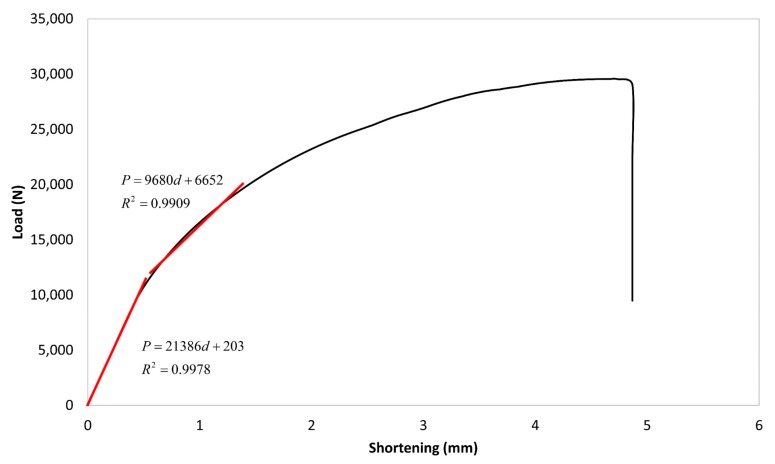
Critical buckling load determination of the current study FEM result (0.1*h* imperfection amplitude).

By using a simple method of straight-line intersection introduced in Debski et al. [20], the critical buckling load could be determined using the curve of the post-buckling equilibrium path. Using this method, the approximation line of pre-buckling and post-buckling path could be linearly determined. Then, the critical load could be obtained by taking the value of the intersection point of those approximation lines. The approximation equations based on the load *P* and shortening *d* are presented in Figure 10 with significantly high determination coefficient (*R*^2^) values. The result showed that the critical buckling load determined by the post-buckling analysis was about 11,589 N, which was 2.7% different from the critical buckling load obtained from the linear buckling analysis (i.e., *P_cr_* = 11,285 N). Therefore, for the purpose of critical buckling load determination, the linear buckling analysis could be used.

## 3. Parametric Analysis and Proposal

This section presents a parametric study of the buckling behavior of the thin-walled GFRP composite strut based on the developed FE model. Different cross-section dimensions and different thickness ratios between the web and flange of the strut were considered. Moreover, the effect of the slenderness ratio on the buckling behavior of the composite strut was also investigated. A cost-effective design of the strut was also studied and proposed. The results provide researchers and designers with a wider vision to select an effective design for their problems.

### 3.1. Optimal Section Dimension

In a practical design, when designing for the buckling resistance of a column subjected to a compressive load, it is necessary to find an appropriate dimension that produces the highest critical buckling load [34,35]. In this section, the optimal dimension for the strut cross-section was found under the conditions of a constant strut thickness (i.e., *t* = 2.08 mm) and a constant cross-section area (i.e., *A* = 332.8 mm^2^). The original strut consisted of two 40-mm flanges and one 80-mm web in terms of length. A dimension factor κ, which describes the flange length/web length ratio, was introduced. The effect of the dimension on the critical buckling load was investigated by varying the dimension factor κ. The buckling mode shape was also an important factor when designing the structure. This was illustrated using the inflection points at which the internal bending moments were zero and the curvature of the structure changed its sign. It showed the possible buckling failure location on the structure such that designers can propose an appropriate solution for strengthening the structure by using stiffeners or other methods [36,37]. Thus, the first buckling mode shape of the strut corresponding to different κ values was investigated and is illustrated in Table 4.

As can be seen in Table 4, the buckling mode shape significantly changed when the dimension factor was varied. Each of them corresponded to a certain buckling resistance capacity. When κ was around 0.167, i.e., the web length was much longer than the flange length, the buckling primarily occurred on the web plate. The web plate became a single load-resisting component with a high slenderness. Thus, its critical buckling load was significantly reduced. The strut had three inflection points in its body. The buckling failure tended to rise at the positions of 1/3 and 2/3 of the length of the strut. In the case where κ=0.3, i.e., the web was about three times longer than the flange, the buckling mode shape included four inflection points or three waves on its body. The buckling failure focused on the center of the web (i.e., 1/2 the length of the strut). However, when κ was equal to 0.452 or higher, the number of waves changed to two, and the buckling on the flange was now more significant than on the web. Obviously, this phenomenon is completely consistent with the theory, because when κ increases, the slenderness of the web decreased while the flange’s slenderness increased. For the results of the buckling mode shape, the researchers and designer may have a more precise design for strengthening the composite GFRP strut.

**Figure 11:**
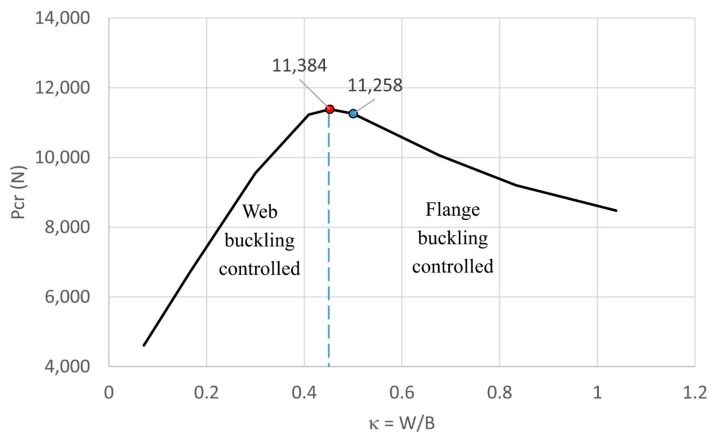
Effect of different dimension ratios on the critical buckling load.

The effect of various dimension factors κ on the critical buckling load is shown in Figure 11. It is interesting to see that when κ<0.452 (i.e., web buckling was controlled), the critical load increased very significantly when κ increased; however, when κ>0.452 (i.e., flange buckling was controlled), the critical load decreased with a lower slope when κ decreased. Obviously, this observation revealed that buckling of the web had a more significant effect on reducing the critical load than the buckling of the flange. It was also noted that, when the original dimension of the web length was 2 times greater than the flange length, the buckling load was 11,258 N. Meanwhile the greatest critical buckling load was 11,384 N, corresponding to κ=0.452, which means the web length was 2.2 times greater than the flange length; however, that increment of the critical load was not very significant. The results provided in this section allow researchers and designers to select an optimal dimension for the strut that will generate the highest critical buckling load.

### 3.2. Optimal Thickness Ratio

In case the dimension of the GFRP strut cannot be changed, it is important to select an appropriate thickness of the web and flange such that the designed strut achieves the highest critical buckling load. In this section, the thickness ratio η between the flange $t_{f}$ and the web $t_{w}$ is introduced. It was varied to investigate the effect of different flange/web thicknesses on the buckling load. The strut dimension, i.e., *B* × *W* = 80 mm × 40 mm, and the cross-section area of 332.8 mm^2^ were held constant. The number of layers, which was eight plies, was also constant. The single thickness of each ply was assumed to be the same for the eight layers. By varying the single thickness of each ply $t_{1}$, the total thickness of the web or the flange will be $t_{1}\times 8$. The flange/web thickness ratio was varied from 0.238 to 2.250. The investigation results revealed an optimal thickness ratio that produced the highest buckling load. The buckling mode shapes corresponding to each thickness ratio are presented in Table 5.

As can be seen, the buckling modes had various shapes when the thickness ratio changed. When the web thickness was high (i.e., η was small) the buckling failure occurred only on the flange and vice versa. Because the slenderness of the web was higher than the flange, the mode shape of the web when η was big contained more waves than the flange one. The greatest buckling load strut was obtained at η=1.363, where its mode shape contained only two waves. According to the buckling mode shape results, researchers and designers can propose an appropriate solution for strengthening a column.

**Figure 12:**
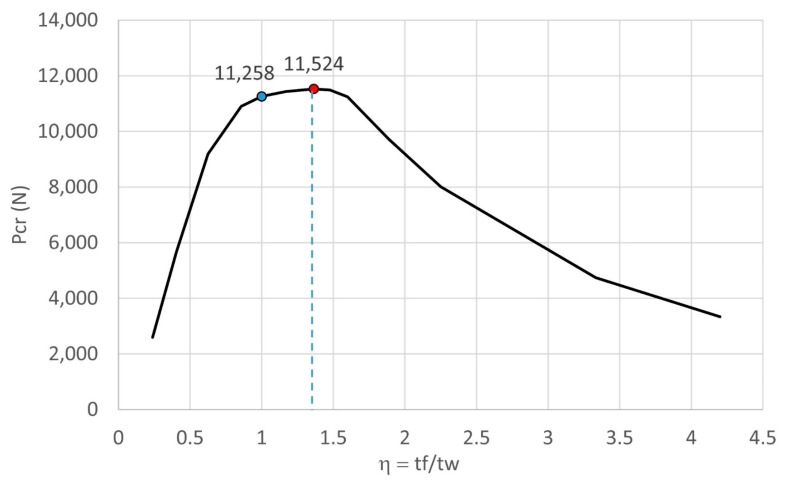
Relation effect of web and flange thickness on critical buckling load.

The effect of the thickness ratio on the critical buckling load is presented in Figure 12. From the graph, the maximum buckling load was obtained at η=1.363 with $P_{cr}$ = 11,524 N. This corresponded to a web thickness of 0.22 mm and a flange thickness of 0.3 mm. The maximum buckling load was 2.36% greater than the original strut with the buckling load of 11 258 N. From the results, the critical buckling load dramatically decreased when η was smaller than the peak point (i.e., η=1.363). In contrast, it only slightly decreased when η was greater than the peak point. This was because the slenderness of the flange was smaller than the web. When the flange thickness increased, it significantly contributed to resisting the compressive load. This resulted in the gradual decrease of the buckling load. The result indicates that to obtain a more stable strut, the thickness ratio η should be in the range of approximately 0.85–1.60. This means the thickness of the flange should not be smaller than 15% or greater than 60% of the web thickness. In addition, the option of selecting the thickness of the flange such that it is greater than the web is recommended.

### 3.3. Cost-Effective Design

When a GFRP strut is produced using the Pultrusion manufacturing method [38], which is a very flexible method, the thickness of the strut can be arbitrary. However, this method cannot produce profile-type products with a high accuracy and a high completion requirements [39]. Thus, to achieve a good production of a GFRP strut, the auto-clave technique can be used. It is a trade-off between flexibility and high accuracy. According to this technique, the thickness of the web and the flange should be the same for cost-effective manufacturing. Therefore, another problem was investigated regarding which thickness of the web and flange is more effective when they are identical to each other.

**Figure 13:**
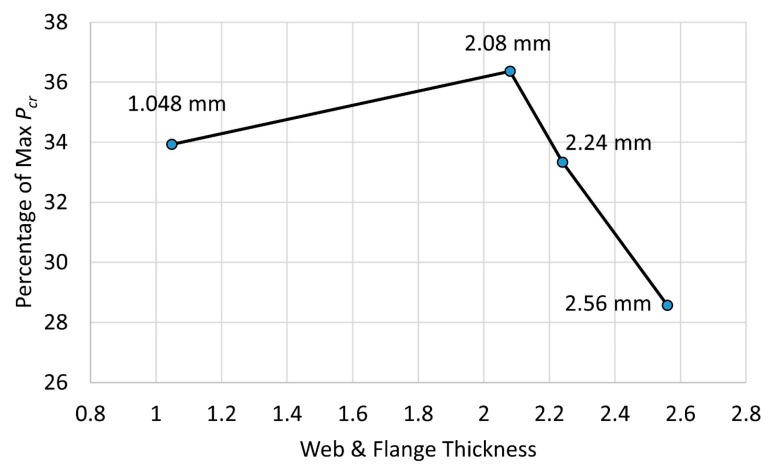
Cost efficiency of column thickness on the critical buckling load.

To find the most cost-effective design, for a certain cross-section area (e.g., *A* = 332.8 mm^2^ for $t_{f}=t_{w}=2.08\;\mathrm{mm}$), a fixed length of 250 mm, and a dimension of *B* × *W* = 80 mm × 40 mm, the investigation of different flange/web thickness ratios following the same manner as Section 3.2 was performed. The result from this investigation provided us with a value of the difference percentage between the optimal ratio at which the maximum critical buckling load was achieved and the original ratio η=1.0. The lower this value was, the more cost-effective the design was. Four thickness types were investigation, which were $t_{f}=t_{w}=1.048\;\mathrm{mm},\;2.08\;\mathrm{mm},\;2.24\;\mathrm{mm},\;2.56\;\mathrm{mm}$. The result of this investigation is presented in Figure 13. As can be seen, the $t_{f}=t_{w}=2.56\;\mathrm{mm}$ was the most effective design case compared to other cases. The thickness, studied in this paper, i.e., $t_{f}=t_{w}=2.08\;\mathrm{mm}$, had a thickness that was 36.4% away from the optimal thickness. These results provided an effective tool to make an appropriate decision when selecting a thickness for the best buckling resistance of a GFRP strut subjected to a compressive load.

### 3.4. Slenderness Ratio Effect and the Proposed Column Curve

In this section, the slenderness ratio effect of the strut on the critical buckling load is presented and the design curve for the GFRP column is proposed. The thickness ratio between the flange and the web was kept at 1.0. To investigate various slenderness ratios, the length of the strut was varied while the cross-section area of the strut was kept constant. For the design purpose, the slenderness ratio effect was considered a non-dimensional quantity $P_{cr}/P_{y}$, where $P_{y}$ is the yield load governing the limit state of the GFRP strut.

The slenderness ratio was calculated as the ratio of the strut effective length over the least radius of gyration of its channel cross-section.

(9) $$\lambda_{c}=\frac{KL}{r_{\min}},$$

where K was equal to 0.65, corresponding to the modified fixed boundary condition of the strut. The effective length KL was selected based on the boundary condition of the experimental setup, which behaved like a fixed condition. However, because of the imperfection condition when setting up the test, the effective length for the fixed condition was recommended to be equal to 0.65 for this design [40]. The radius of gyration of the channel section strut was calculated using $r_{\min}=\sqrt{\frac{I_{y}}{A}}$, where *I_y_* is the least area moment of inertia (i.e., y axis in this case), and A is the area of the GFRP strut with the flange × web dimension of *W* × *B* = 40 mm × 80 mm and the thickness of $t_{f}=t_{w}=2.08\;\mathrm{mm}$.

The critical buckling load $P_{cr}$ was obtained from the numerical analysis of the FE model. The yield load $P_{y}$ is expressed as:

(10) $$P_{y}=\sigma_{y}A,$$

where $\sigma_{y}$ denotes the compressive yield stress of the GFRP material.

**Figure 14:**
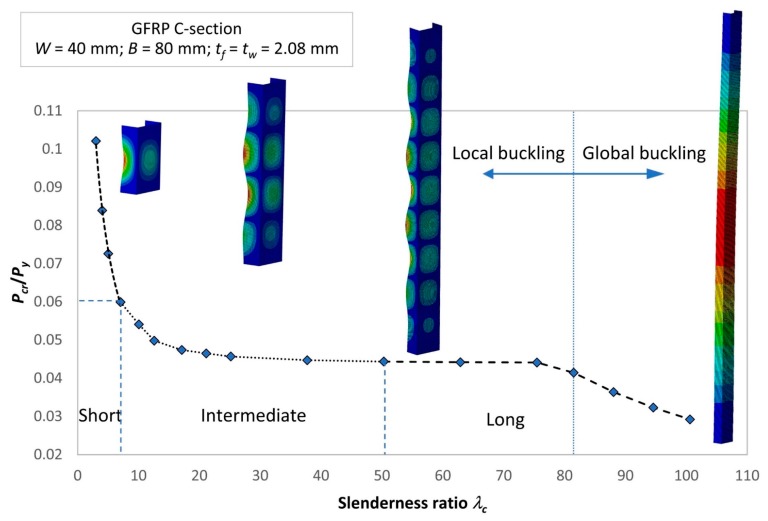
Design curve for the GFRP channel section column.

The slenderness ratio effect on the critical buckling load is illustrated in Figure 14. The interaction graph is presented as a design curve for a GFRP strut, including the value of $P_{cr}/P_{y}$ and buckling results of the corresponding strut. It should be noted that the slenderness ratio was investigated in the range of $P_{cr}=\left[0.03P_{y},\;0.1P_{y}\right]$. As can be seen, for the short strut, the critical buckling load dramatically decreased when the slenderness ratio $\lambda_{c}$ increased from 3.0 to 7.0. The intermediate strut and long strut was assigned in the slenderness ratio range of $\lambda_{c}=\left[7.0,\;50.0\right]$ and $\lambda_{c}=\left[50.0,\;100.0\right]$, respectively. Their critical buckling loads slightly decreased when the slenderness ratio increased. The value of $P_{cr}/P_{y}$ shows that the yield load was much higher than the buckling load, which means the thin-walled GFRP strut mainly failed due to the buckling rather than the yield limit being exceeded.

Based on the obtained $P_{cr}/P_{y}$ results, the following design equations for the GFRP strut were proposed by using a regression technique.

(11) $$\frac{P_{cr}}{P_{y}}=\left\{ \begin{array}{lll} 0.2029{\lambda_{c}}^{-0.63} & \left(R^{2}=0.998\right) & if\;3.0\leq \lambda_{c}\leq 7.0\\ 0.045+0.052e^{-0.174\lambda_{c}} & \left(R^{2}=0.995\right) & if\;7.0<\lambda_{c}\leq 50.0\\ 0.028+\frac{0.016}{1+{\left(\lambda_{c}/88.67\right)}^{17.94}} & \left(R^{2}=0.997\right) & if\;50.0<\lambda_{c}\leq 100.0 \end{array}\right.,$$

As can be seen, the determination coefficient (*R* squared) values are very close to 1.0, showing that the proposed equations fit the obtained experimental results. These equations are first proposed for designing the GFRP strut. Based on them, researchers or designers are able to calculate the critical buckling load according to the dimensions of the GFRP strut, which are expressed through the slenderness ratio. In addition, the results in Figure 14 show that local buckling occurred when the slenderness ratio was smaller than 81.5; however, it began to behave as global buckling when the slenderness ratio was greater than 81.5. This demarcation point provides researchers and designers with a clearer observation of the behavior of the strut when the slenderness is changed. Thus, a more precise design can be obtained to increase the stability of the strut.

## 4. Conclusions

This study provided a significant and useful investigation based on a numerical study of the dimension effect on buckling behavior of a thin-walled GFRP composite strut. A reliable finite element model of the GFRP strut under compressive loading was developed. A parametric study was conducted to investigate the effect of the slenderness ratio on the critical buckling load. The following conclusions were achieved:

(1) The maximum critical buckling load was obtained when the length of the web was around 2.2 times greater than the flange length. The two-wave buckling mode shape, which contained three inflection points, withstood a higher buckling load.
(2) When the thickness ratio between the web and the flange stayed in the range of 0.85–1.60, a good buckling load could be withstood.
(3) A cost-effective design could be obtained when the thickness of the web and flange of $t_{f}=t_{w}=2.56$ mm was used.
(4) The buckling behavior of the GFRP strut changed from local buckling to global buckling at a demarcation point with a slenderness ratio of λ=81.5.
(5) The proposed design curve and equations for the GFRP strut provided a precise method for calculating the critical buckling load based on the input dimensions of the GFRP strut.

This study is limited to the quasi-isotropic laminate material and only the elastic buckling behavior. In further research, the design curve of many different types of laminates that considers post-buckling and imperfection buckling behaviors can be employed.

## Figures and Tables

**Table 1:** Laminate ply Thickness.

Ply	1 [90°]	2 [45°]	3 [−45°]	4 [0°]	5 [0°]	6 [−45°]	7 [45°]	8 [90°]
Thickness (mm)	0.26	0.26	0.26	0.26	0.26	0.26	0.26	0.26

**Table 2:** Material Properties of the GFRP Strut Used for the Simulation.

$E_{1}\;(\mathrm{MPa})$	$E_{2}\;(\mathrm{MPa})$	$v_{12}=v_{21}$	$G_{12}\;(\mathrm{MPa})$	$G_{23}\;(\mathrm{MPa})$	$G_{13}\;(\mathrm{MPa})$
38,000	81,000	0.27	2000	2000	2000
$X^{T}$ (MPa)	$X^{C}$ (MPa)	$Y^{T}$ (MPa)	$Y^{C}$ (MPa)	S (MPa)	
792	679	39	71	102	

where *X^T^* and *X^C^* denote the tensile and compressive strengths in the fiber direction; *Y^T^* and *Y^C^* denote the tensile and compressive strengths in the matrix direction; and S includes *S^L^* and *S^T^*, which denote the longitudinal and transverse shear strengths, respectively.

**Table 3:** Comparison of the Critical Buckling Load between the Numerical and Experimental Results.

Laminate Types	Critical Buckling Load (N)	
	Numerical Results	Experiment Results
Quasi-isotropic laminate [0/−45/+45/90]s	11,285	10,500 ± 10%
Angle-ply laminate [+45/−45/+45/−45]s	12,652	11,700 ± 10%

**Table 4:** First Buckling Modes of the Strut with Different Flange/web Dimension Ratios.

***κ*** = ***W*/*B***	0.167	0.300	0.452	0.500	1.038
***P_cr_* (N)**	6727.8	9556.9	11384	11258	8476.2
**First Buckling Mode**	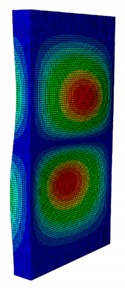	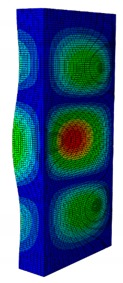	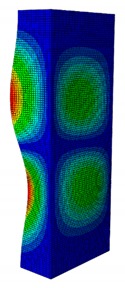	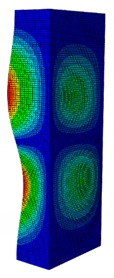	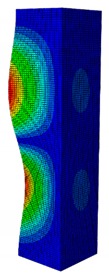

**Table 5:** First Buckling Modes of the Strut with Different Flange/web Thickness Ratios.

$\eta =t_{f}/t_{w}$	0.238	0.405	0.625	0.857	1.000
***P_cr_* (N)**	2595.8	5678.5	9190.1	10,904	11,258
**First Buckling Mode**	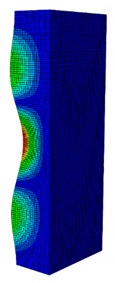	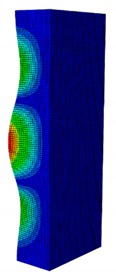	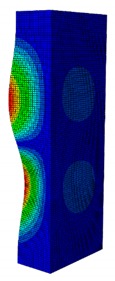	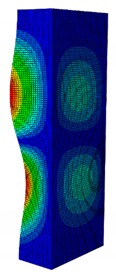	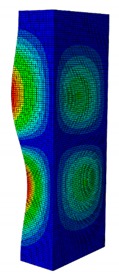
$\eta =t_{f}/t_{w}$	1.363	1.476	1.600	1.889	2.250
***P_cr_* (N)**	11,524	11,492	11,244	9714.3	8009.3
**First Buckling Mode**